# A tool to assess the internal validity of toxicokinetic studies

**DOI:** 10.1007/s00204-026-04387-y

**Published:** 2026-04-15

**Authors:** M. Batke, G. Damm, H. Foth, A. Freyberger, J. G. Hengstler, A. Mangerich, H. Mielke, F. Partosch, T. Schupp, A. Sonnenburg, J. vom Brocke, K.-M. Wollin, E. A. Algharably, U. Gundert-Remy

**Affiliations:** 1 Faculty of Chemistry, University of Duisburg-Essen, Essen, Germany; 2 Department of Hepatobiliary Surgery and Visceral Transplantation, Leipzig University Medical Center, Leipzig, Germany; 3 Institute of Environmental Toxicology, University of Halle, Halle/Saale, Germany; 4 Formerly Bayer AG, Wuppertal, Germany; 5 Leibniz Research Centre for Working Environment and Human Factors, Dortmund University, Dortmund, Germany; 6 Nutritional Toxicology Group, University of Potsdam, Institute of Nutritional Science, Potsdam, Germany; 7 Department of Exposure, German Federal Institute for Risk Assessment, Berlin, Germany; 8 Ramboll Deutschland GmbH, Essen, Germany; 9 University of Applied Sciences Muenster, Steinfurt, Germany; 10 Department Pesticides Safety, German Federal Institute for Risk Assessment, Berlin, Germany; 11 European Chemicals Agency, Helsinki, Finland; 12 Formerly Public Health Agency of Lower Saxony, Hannover, Germany; 13 Charité-Universitätsmedizin Berlin, Freie Universität Berlin and Humboldt Universität Zu Berlin, Berlin, Germany

**Keywords:** Systematic review, Animal toxicokinetics, Toxicokinetic assessment tool, Internal validity

## Abstract

Toxicological animal studies are essential to risk assessment. The reliability of the studies depends on the extent to which study design, conduct, and analysis have a high internal validity. Several tools exist for assessing the internal validity of human and animal studies, however, a dedicated tool for animal toxicokinetic studies is lacking. We developed a questionnaire to systematically assess toxicokinetic animal studies. The questionnaire consists of seven main domains with questions for which points are given by the assessor multiplied by fixed weighting factors (WF). The scores are then aggregated at both the domain and overall levels and expressed as a percentage of the maximum score. We established four confidence tiers based on the percentage of the achieved score. Experts were able to discriminate studies based on internal validity (overall assessment rating between 37.6% and 86.9%, n = 7 studies). The questionnaire was also a helpful tool for less experienced scientists (master students, postgraduates) helping them to assess the internal validity in a systematic way. By implementing a WF into the calculation and performing the assessment at the domain level, both innovative steps in the assessment process, the transparency of the assessment is increased. In a limited exploratory study, we found that Large Language Models (AI) were able to complete the questionnaire, however, failing to discriminate studies’ quality. This newly developed questionnaire provides a transparent, granular, and effective framework for assessing the internal validity of animal toxicokinetic studies, offering reliable discrimination across quality tiers for both experienced and toxicologists in training.

**Supplementary Information:**

The online version contains supplementary material available at 10.1007/s00204-026-04387-y.

## Introduction

Toxicological animal studies represent a critical component of risk assessment which increasingly utilizes systematic review methodologies that have a long-standing tradition in the medical field (Chalmers 1993). Originally developed to evaluate the effectiveness of clinical interventions and diagnostic procedures, these frameworks have been continuously refined by the Cochrane Collaboration. Systematic review principles have been extended to further fields and are also applied in chemical risk assessment, supporting transparent analysis of animal and human studies. In the risk assessment of substances such as e.g. food additives, plastics, or antioxidants, systematic review principles are applied by national and international agencies such as US Environmental Protection Agency (EPA 2022), and the European Food Safety Authority (EFSA FAF Panel 2021; Gundert-Remy et al. 2017). Quality assessment of a study refers to the methodological quality (so called internal validity or risk of bias). Several domains are evaluated including the design, conduct, and reporting of a study, the assessment of which reveals the extent a domain may introduce bias. To date, tools exist for the systematic assessment of the internal validity of human and animal studies. For example, for human studies: Cochrane Risk of Bias Tool, ROBINS-I (Higgins JPT 2016), Jadad scale (Clark et al. 1999), Newcastle–Ottawa Scale (Gualdi-Russo and Zaccagni 2026) and the NTP-OHAT approach (NTP-OHAT 2015, 2019); for animal studies: SYRCLE (Hooijmans et al. 2014), NTP-OHAT (NTP-OHAT 2015, 2019) and SciRAP (Beronius et al. 2018). Soliman et al. (2022) have published quality markers for human pharmacokinetic studies. Existing animal risk-of-bias tools do not capture key toxicokinetics-specific validity issues. Notably, international guidelines related to toxicokinetics (ICH 2022; OECD 2010) deal with the aspect of internal validity but only to a limited extent. Similarly, there is a EMA guidance document concerning the conduct of animal pharmacokinetic studies, but, it does not serve as direct quality assessment of published animal kinetic studies (EMA 2017). Hence, a dedicated tool to assess internal validity in animal toxicokinetics studies is lacking to date. Developing a fit-for-purpose tool that integrates both animal-study and kinetic-specific validity domains is therefore essential to improve the interpretability and comparability of animal toxicokinetics research.

In this work, we describe the development and the items of a questionnaire intended for use as a tool for quantitatively assessing the internal validity of a toxicokinetic animal study as part of a systematic review. We also describe results of testing the questionnaire relating to (i) its discriminatory capacity to separate studies based on internal validity, (ii) the expertise necessary to handle the questionnaire by comparing the results of assessment in groups with differing experience in toxicology and (iii) the ability of available AI tools to discriminate between the internal validity of studies using the questionnaire.

## Methods

### Development of the questionnaire

A literature review was performed to identify examples for domains and preliminary questions from other questionnaires developed for systematic assessment of the internal study validity (Cooper et al. 2016; EFSA FAF Panel 2021; EPA 2022; Gundert-Remy et al. 2017; Higgins et al. 2024; Mathisen et al. 2025; NTP-OHAT 2015; NTP-OHAT 2019; Roth et al. 2021; Soliman et al. 2022; Sterne et al. 2019).

From the compilation of material found in the literature review, several domains and questions were selected and a structured consensus process was applied by conducting a Delphi-like survey (Dalkey and Helmer 1963) by mail, as well as panel discussions after every round (online, n = 8; in-person, n = 3) until consensus was reached. The participants were experienced toxicologists who are members of the standing advisory committee of the German Society of Toxicology (see authors with the exception of EAA), with ample practical experience in performing systematic reviews in the field of toxicology; additional in-depth expertise was available in the field of chemical analytics, bioanalytics as well as toxicokinetics and toxicokinetic modelling. Explaining texts were developed by the experts for every question as a support to understand what the question addressed, utilizing the same approach as the National Toxicology Program Office of Health Assessment (NTP-OHAT 2015, 2019).

Participating experts discussed and reached a consensus on the scores for each question. To ensure the assessment accurately reflected the relative significance/relevance of each question, weighting factors were integrated into this same process to account for the varying impact of specific question rating on the overall outcome.

Each question’s score was multiplied by its respective weighting factor to yield the corresponding number of points. The number of points for the questions were summed up for every domain. The sum of points per domain was calculated and expressed as percentage of the maximum attainable points (i.e., the number of points if the highest scores would have been assigned to each question). In the calculation, we also took into consideration that a possible answer could be “not applicable, n.a.” which reduces the potential total. Consequently, the final study assessment was determined by expressing the sum of points for each domain as a percentage of these adjusted maximums using the following formula:

$$Domain\; Score \left( \% \right) = \frac{{\Sigma po{\mathrm{int}} s\; of\; all\; questions}}{{\max attainable\; po{\mathrm{int}} s \;\left( {adjusted \;for\; n.a.} \right)}} \times 100$$

After finalizing the questionnaire, the results of the calculation are displayed in a separate table (Online Resource 1). The final decision on the internal validity is used for an overall assessment of the study for which tiers were allocated as performed in other areas of quality assessment e.g. NTP-OHAT (2015); NTP-OHAT (2019). We established four confidence tiers based on the percentage of maximum reachable points achieved in the overall assessment: very low (0–25%), low (> 25–50%), moderate (> 50–75%), and high (> 75%).

### Testing the questionnaire for its discriminatory ability

To test the questionnaire’s ability to discriminate between studies with differing internal validity, members of the committee (“the experts”) selected studies by consensus. These had high, medium and low internal validity according to their personal evaluation by a non-systematic approach without applying a standardized procedure. The studies were then assessed applying the questionnaire by the experts to find out whether the questionnaire had the potential to discriminate between studies with differing internal validity. The following studies were selected: (AnandaKumar et al. 2025; Gidez and Karnovsky 1954; Howes et al. 1998; Humphreys and Triffitt 1968; Kwon et al. 2024; Michael and Coots 1971; Roberts and Renwick 2008).

### Testing the questionnaire for applicability in groups with differing toxicological expertise

To evaluate the influence of the level of expertise in toxicology on the response to the questions in the questionnaire, two publications (AnandaKumar et al. 2025; Kwon et al. 2024) were selected. The publication of Kwon et al. (2024) was selected because the study investigates nanoparticulate forms of SiO_2_ for which particular attention has to be paid to documentation of purity and formulation according to current requirements (EFSA Scientific Committee 2021). The questionnaire was filled out by groups with differing experience in toxicology: (i) Master students of toxicology, and (ii) attendees of a postgraduate toxicology course (not all trained toxicologists); and their results were compared to the results of more experienced toxicologists (members of the standing advisory committee of the German Society of Toxicology, the “experts”, n = 6). The two publications were chosen so that they both were in the range between > 66 and 100% when assessed by the experts. Eight Master students and 22 postgraduate attendees assessed Kwon et al. (2024); 13 Master students and 14 postgraduate attendees assessed the study of AnandaKumar et al. (2025). The number of participants was determined by the number of students in the courses willing to participate.

### Exploratory study on the discrimination ability of some AI tools using the questionnaire

Furthermore, several Large Language Models—commonly called “Artificial Intelligence” (AI) (ChatGPT Thinking 5.1, DeepSeek R1 0528, MedGemma 27B Instruct, Meta Llama 3.1 8B Instruct, Perplexity Deep Research) were tested in a limited exploratory exercise to evaluate their capacity to apply the questionnaire, consequently, to discriminate between the internal validity of studies. The same studies were used as those for testing the discriminatory ability of the questionnaire. The following unified prompt was used “I have a scientific paper. I want you to answer a couple of questions on the article. Please answer with scores: n.a.- not applicable; 0—not reported; 1—major flaws; 2—acceptable with restrictions; 3—acceptable. I upload the article in pdf format and the questions in an excel table. In the first column, the domain of the question is specified. In the second column, you will find the questions, in the third column there is some explanation on the question. Give your score in column 4 and a justification (one or two sentences) for your score in column 5. All questions relate to the article. Provide the result as an excel file for download. Work on your own, do not ask anything before you are finished.”

The selection of AI tools was not systematic and did not aim to be comprehensive, but was based on their accessibility.

### Data analysis

Data analysis and graphical presentation were performed with R Project for Statistical Computing, version 4.5.1(R Core Team 2025). Comparisons for the discriminatory ability and questionnaire application among groups with differing expertise was done at the level of overall study assessment and at the level of domains. Differences between levels of expertise in toxicology were analyzed using Kruskal–Wallis non-parametric variance analysis with and without Bonferroni adjustment for multiple testing. The data were presented as Box plots showing median and 25th and 75th percentile.

## Results

### Development of the questionnaire

The final questionnaire (Table 1) comprised an overall evaluation and six domains with a total of 23 questions to be answered. The domains were: (1) Study Design and Planning, (2) Identity, Purity of the test substance (3) Formulation of the test item, (4) Study Conduct, (5) Sample Collection and Analysis (sampling and analysis) (6) Toxicokinetic analysis and data reporting. The number of questions was 1 for Study Design and Planning, 2 for Purity and Formulation, 3 for Formulation of the test item, 7 for Study Conduct, 5 for Sample Collection and Analysis, 5 for Toxicokinetic analysis and data reporting. The following scores were applied to every question: 0 = not reported; 1 = major flaws; 2 = acceptable with restrictions; 3 = acceptable. If the question was not appropriate (e.g. the intention was not to measure the concentration–time- profile in the plasma), then it was rated as not applicable (n.a.). Each score was multiplied by a weighting factor (WF) which ranged from 1 to 4 reflecting the importance of the question.

**Table 1:** Questionnaire to assess the internal validity of toxicokinetic studies in animals

Domain	Elements considered in the assessment as Category and subcategory	Explanation	Scoring ^a^	Weighting factor
1. Study design and planning	a. Were study objectives clearly defined?	Authors should provide a clear statement of the objectives of the research to clarify the purpose and the scope of the study		2
2. Identity, purity	a. Was the test substance clearly identified? Was a chemical identifier given, e.g. CAS number?	Unique identifier (e.g. IUPAC name, CAS- No, molecular structure) If applicable, information on isomers; If the test substance is labelled, information on the following should be included in this subsection: the type of label, position of label, and in case of radiolabel specific activity. Substances synthesized in-house or provided by a sponsor have to be qualitatively identified by an appropriate analytical method		1
	b. Was the purity of the applied substance given? Were impurities identified?	Numerical specification of chemical purity and if applicable, radiochemical purity shall be provided. When chemical purity is below 90%, identification of any impurities For substances with a purity > 90% and with clear source information (e.g. supplier and Lot.no.), statement of purity value is sufficient for score 3. For substances synthesized in-house or provided through a sponsor, quantitative determination of its chemical composition by a relevant analytical method should be described		1
3. Formulation of test item	a. Was the tested formulation clearly described?	The type or description of any vehicle, diluents, suspending agents, and emulsifiers or other materials used in administering the test substance should be stated. The concentration of test item in the formulation should be stated		3
	b. Was the application formulation adequately prepared e.g. was the test item appropriately dispersed or dissolved in the formulation?	Ideally, the test item should be soluble in the vehicle and should not undergo any reactions. If the test item is not soluble in any appropriate vehicle, a homogeneous dispersion should be achieved, with nano particles an adequate pretreatment is necessary		3
	c. Are the stability and homogeneity of the test item in the formulation verified with appropriate analytical methods? Was the concentration of the test substance in the formulation measured with appropriate methods?	If stability cannot be demonstrated for the duration of the treatment period, procedures applied to deal with less stable test items should be described in detail, such as daily preparation of the application formulation		3
4. Study conduct	a. Were test animal species and strain clearly described?	Information provided on the test animals, including selection and justification for species (The choice of species should consider factors like metabolism, excretion pathways and size), strain, and age at study initiation, sex as well as body weight, health status		1
	b. Was the number of animals per treatment group adequately chosen?	A minimum of four animals per sex per dose group is to be used. If only one sex is used the number of animals should be increased to reach eight animals. The number of animals used should be stated with materials and methods part		2
	c. Was the number of dose groups adequately chosen and justified?	The number of dose groups depends on the study objective. Evaluating for nonlinearity requires multiple dose levels		2
	d. Were dosage levels justified?	Justification for selection of dose levels based on previous data or relevant literature. Dose setting should be determined by the nature of the experiment and/or the issue being addressed		1
	e. Administration route	Justification for route of administration: Were the route(s) and methods of administration/exposure clearly stated and appropriate for the study objective (oral gavage, intravenous injection, consideration of application volume etc.)?		3
	f. Was the dosing regimen stated (single vs. repeated dosage)?	The frequency of dosing should be determined by the nature of the experiment and/or the issue being addressed		4
	g. Are housing conditions reported? If relevant, were measures taken to avoid housing- or handling-related exposure to substances that could interfere with the test results?	If housing conditions are referred to as standard, respective guidelines or protocols (e.g. SOPs) should be cited and included in the reference list		2
5. Sample collection and analysis	a. Were sufficiently sensitive and specific bioanalytical methods for analyte quantification used and described?	Were the used biological sample analytical methods described or citations of prior validation studies provided in the publication or affiliated appendix? Are specificity, recovery, LOQ and LOD, linearity, the stability of the assay and its reproducibility described? Was the method sufficiently sensitive for quantification of concentrations following the lowest dose? Refer to EMA/FDA guidelines for bioanalytical method validation		2
	b. Were samples analyzed for individual animals or pooled from several animals?	Pooled samples should be clearly identified and an explanation for pooling provided. (Acceptable individual data; Acceptable with restrictions pooled samples with explanation; If not major flaws;)		3
	c. Was sampling appropriate to answer the research question?	Depending on the aim of the study blood, urine and/or feces have to be collected in appropriate intervals and over an appropriate time period		3
	d. Was the sampling site for blood sample(s) given?	Sampling site should be consistent for all test animals		1
	e. Were sample preparation and stability under storage conditions appropriate and described in a manner that could be accurately replicated?	Sample preparation (anticoagulants, stabilizers, centrifugation), storage, temperature, use of spiked samples		1
6. Toxicokinetic analysis and data reporting	a. Were concentration–time data provided?	Were concentration–time data in the analyzed matrix provided graphically or numerically? (Acceptable: numerical data; Acceptable with restriction: graphical presentation only)		2
	b. Were the equations used to calculate the intended toxicokinetic parameters (e.g. C_max_, T_max_, AUC, t_½_, clearance) presented or cited?	If applicable, was there a clear description of the toxicokinetic model, its development, validation and justification for use? It is recommended to provide the following details about the selected modelling process:—Description of studies from which dataset was driven;—Model structure;—Validated software for the pharmacokinetic analysis;—Criteria for accepting valid model’s parameters;—Fitting procedure defined prior to the initiation of the analysis;—A reasonable assumption based on which the scheme for weighting is considered to be appropriate and the transformation of data [e.g. logarithmic transformation to achieve the homoscedastic (constant) variance requirements] should be provided		2
	c. Were the data reported which were intended to investigate in the study? E.g. absorption, distribution in tissues, metabolism and excretion; were excreted test substance/metabolites quantified and matched against the total dose?	e.g. Rate and extent of absorption of the test substance after administration by the relevant route(s) of exposure; e.g. quantity and percent recovery in urine, faeces, expired air, and urine and faeces cage wash Tissue distribution reported as percent of administered dose and concentration (microgram equivalents per gram of tissue), and tissue-to-blood or tissue-to-plasma ratios; Quantities of the test substance and metabolites (reported as percent of the administered dose) collected in excreta; Reference to appendix data which contain individual animal data for all measurement endpoints (e.g., dose administration, percent recovery, concentrations, toxicokinetic parameters, etc.);		3
	d. Was centrality and variance of the toxicokinetic results presented?			2
	e. Were all animals included in the study accounted for?	Animals which are not included (attrition) should be accounted for and reasons given (e.g. In-/Exclusion criteria, Outlier analysis)		4
7. Overall evaluation	a. Was the chosen study design appropriately selected to answer the study objectives?			4

The questionnaire with the corresponding calculation algorithm are given in Supplementary file 1.

^a^Scoring: n.a.- not applicable; 0—not reported; 1—major flaws; 2—acceptable with restrictions; 3—acceptable.

The most important questions were under (i) formulation of test item, description of formulation, preparation of formulation, stability and homogeneity of formulation (WF3), under (ii) study conduct, the route of administration (WF 3) and the dosing regimen (WF 4), under (iii) sample collection and data analysis (sampling and analysis), information on whether pooled samples or single samples were used (WF 3) and whether appropriate sampling intervals have been applied (WF 4), under (iv) toxicokinetic analysis and data reporting, whether all intended endpoints were reported including quantification of excretion (WF 3), whether the number of reported results match with the number of animals in the study (attrition rate) (WF 4). Finally, in the overall evaluation of the study, whether the design was appropriate to answer the questions (WF 4).

### Testing the questionnaire for its discriminatory ability

**Fig. 1:**
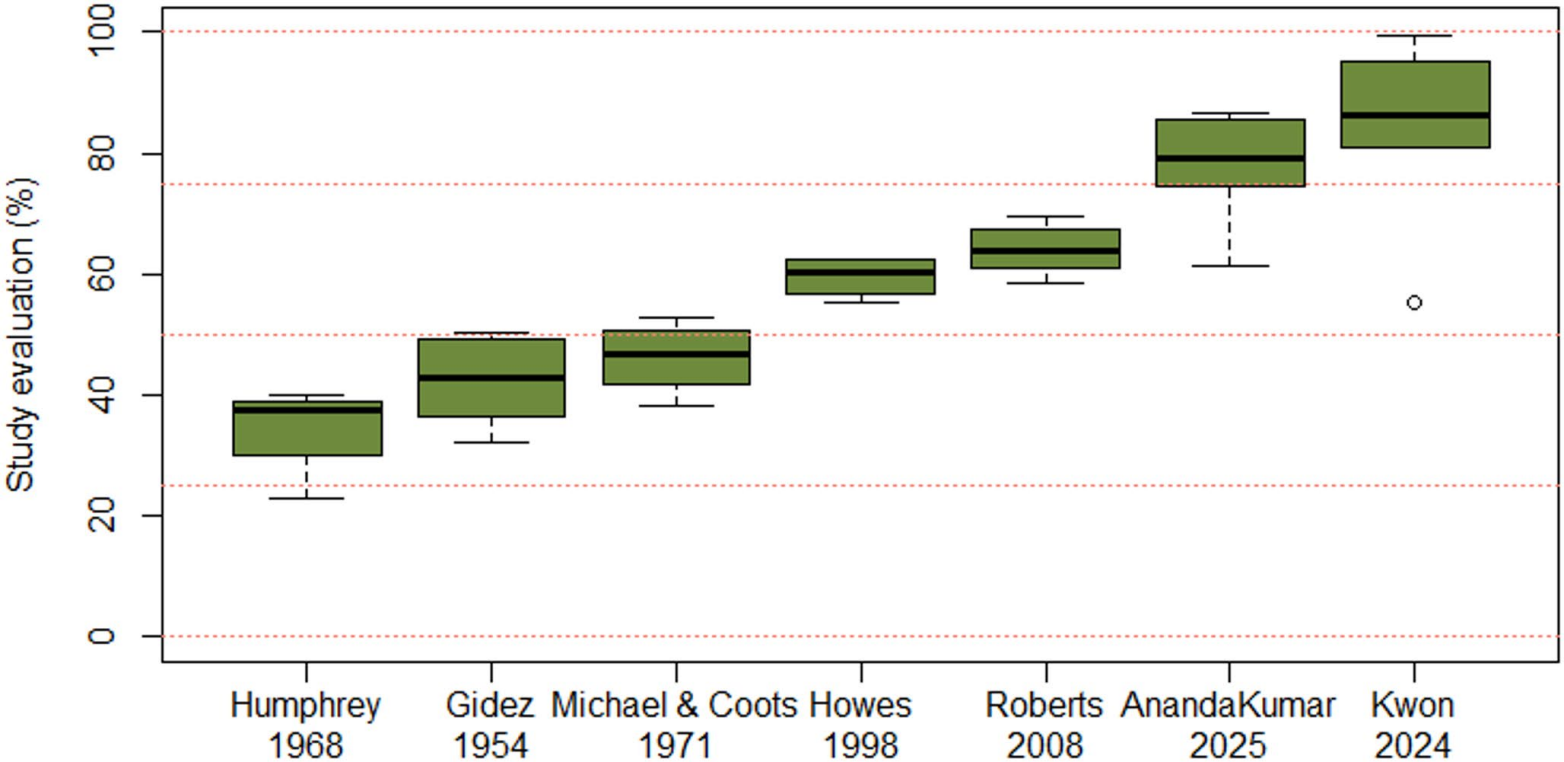
Overall assessment of the selected studies by experts (n = 6). Box-and-whisker plots show the median (horizontal line), 25–75th percentiles (box), and the range of data within 1.5 × the IQR (whiskers). Individual dots represent outliers. Dotted lines show the tiers of confidence. 0–25% = very low confidence; > 25–50% low confidence; > 50–75% moderate confidence; > 75% high confidence

As illustrated in Fig. 1, the expert assessments allocated the seven studies across three confidence strata (i.e. > 25% to 50%, > 50% to 75% and > 75% to 100% of the maximum reachable points). Three studies fell into the low confidence tier (> 25% to 50%), while the remaining four were split evenly between moderate (> 50–75%) and high confidence (> 75–100%) based on their percentage of maximum reachable points.

The results indicate that the questionnaire is capable of discriminating between studies with differing internal validity. Notably, the ranking of the studies corresponds approximately with the publication year of the studies.

Studies classified as having high confidence in their internal validity reached higher percentages of the maximum reachable points for all domains as compared to the other studies. In particular, this is valid for the domains “formulation” and “sampling and analysis” which is not astonishing since we noted that the ranking increases with the publication year and analytical requirements and how to treat the formulation before application were only recently published (EFSA Scientific Committee 2021; ICH 2022) (Table 2).

**Table 2:** Experts’ overall assessment and domain assessment of the selected studies

	Humphreys and Triffitt (1968)	Gidez and Karnovsky (1954)	Michael and Coots (1971)	Howes et al. (1998)	Roberts and Renwick (2008)	AnandaKumar et al. (2025)	Kwon et al. (2024)
Overall assessment	37.6 (33.5–38.6)	42.8 (36.5–49.1)	46.6 (43.4–49.3)	60.3 (57.4–62.4)	63.9 (61.9–66.1)	79.1 (75.2–84.4)	86.3 (82.1–93.2)
Overall study design	61.1 (52.8–66.7)	66.7 (55.6–77.8)	66.7 (55.6–77.8)	72.2 (63.9–77.8)	83.3 (63.9–100)	88.9 (77.8–100)	100 (83.3–100)
Purity & identity	25 (16.7–33.3)	16.7 (16.7–16.7)	33.3 (29.2–33.3)	33.3 (33.3–37.5)	83.3 (79.2–87.5)	83.3 (58.3–83.3)	66.7 (66.7–79.2)
Formulation	27.8 (19.4–33.3)	22.2 (11.1–22.2)	22.2 (19.4–25)	55.6 (47.2–58.3)	22.2 (8.3–36.1)	66.7 (50–66.7)	88.9 (80.6–97.2)
Study conduct	52.2 (40.6–57.8)	53.3 (48.9–60)	66.7 (66.1–68.3)	74.4 (72.2–75.6)	86.7 (80–92.2)	85.6 (78.3–91.1)	90 (85.6–94.4)
Sampling & analysis^a^	25.4 (23.2–27.5)	40.6 (38.1–47.8)	39.4 (31.8–44.4)	54 (50.2–57)	59.4 (57.5–62.3)	81.2 (70.3–90.9)	82.8 (75.4–93.1)

Values represent the median (interquartile range) of the total score calculated as percentage of the maximum reachable points.

^a^ combined analysis of domains 5 and 6 i.e. Sample collection and analysis; Toxicokinetic analysis and data reporting.

### Testing the questionnaire for applicability among groups with differing toxicological expertise

**Fig. 2:**
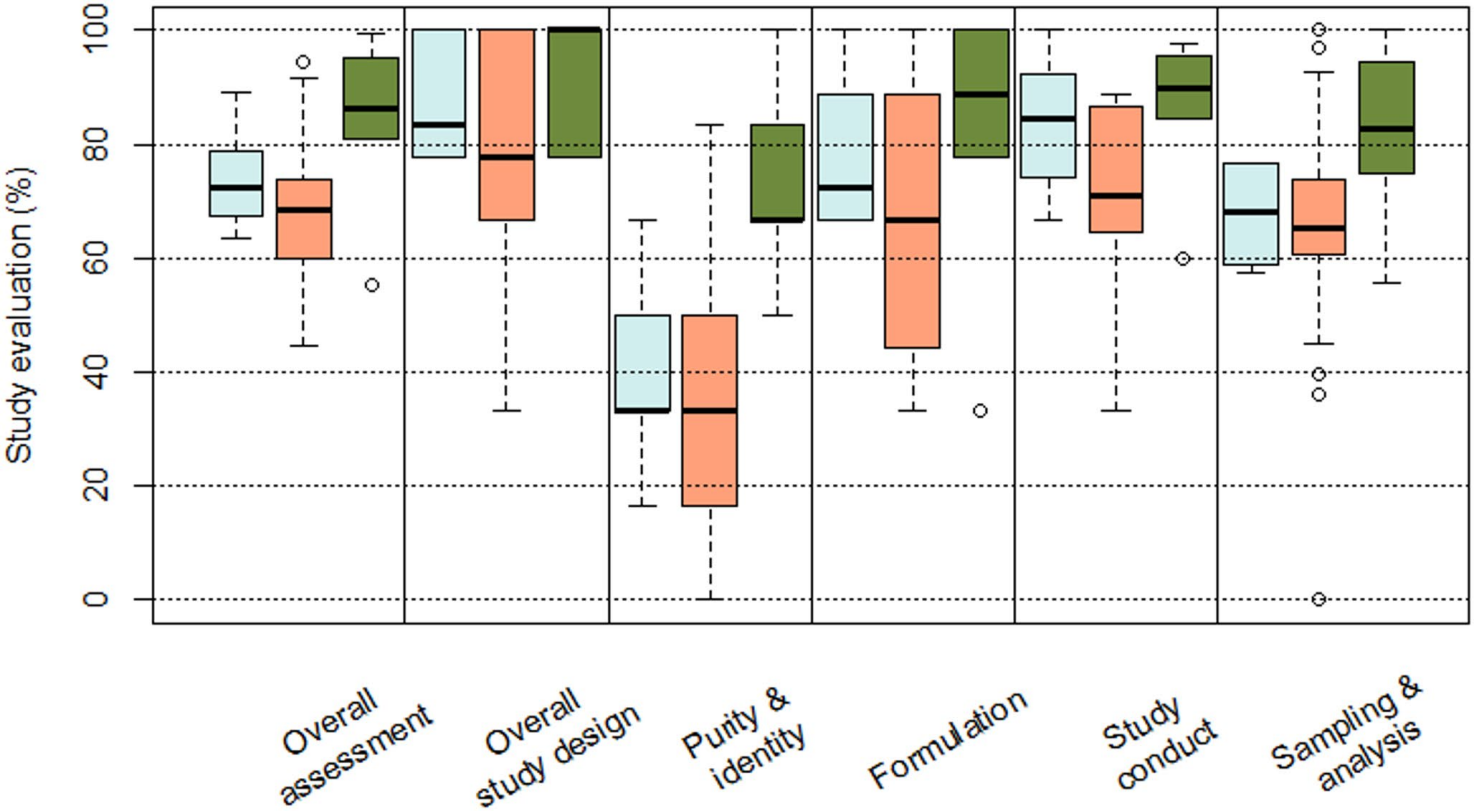
Domain and the overall study assessment for the internal validity of Kwon et al. (2024) using the questionnaire among participating groups. Study evaluation given as percent reached form maximum reachable. Box-and-whisker plots show the median (horizontal line), 25–75th percentiles (box), and the range of data within 1.5 × the IQR (whiskers). Light blue: students of Master course in toxicology (n = 8), salmon-coloured: postgraduate course attendees (n = 22), olive-green: experts (n = 6)

The assessment of a high-confidence tier study (Kwon et al. 2024) by Master students, toxicology postgraduate attendees, and experts (Fig. 2 and Table S1) indicates a difference in the study overall assessment between the three groups which however was not statistically significant (p = 0.063).

The differences are statistically significant (p < 0.05) only for the domains “purity and identity”, “study conduct”, and “sampling and analysis” without Bonferroni correction from multiple testing (Table S1). Notably, in these specific domains, Master’s students and postgraduate participants assigned lower number of points than the experts. With correction for multiple testing none of the results are statistically significant (p > 0.083).

**Table 3:** Overall assessment of studies by selected AI models

Study AI model	Humphreys and Triffitt (1968)	Gidez and Karnovsky (1954)	Michael and Coots (1971)	Howes et al. (1998)	Roberts and Renwick (2008)	AnandaKumar et al. (2025)	Kwon et al. (2024)
Perplexity	81.2	69.7	82.7	90.3	77.0	88.5	93.9
DeepSeek	59.3	69.1	64.8	71.2	69.7	83.0	90.1
MedGemma	70.9	92.5	70.9	70.3	92.7	96.4	96.4
MetaLlama	100	100	99.3	100	100	98.8	89.7
ChatGPT Thinking	73.3	74.2	76.1	83.6	98.2	85.5	82.4
*Experts’ median*	*37.6*	*42.8*	*46.6*	*60.3*	*63.9*	*79.1*	*86.3*

The numbers give the percentage of the scores reached from the maximal reachable scores

For comparison, the experts’ median is shown.

**Fig. 3:**
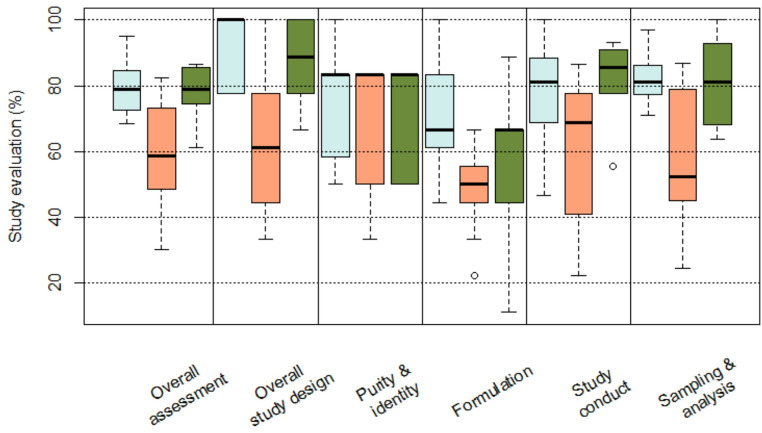
Domain and the overall study assessment for the internal validity AnandaKumar et al. (2025) using the questionnaire among participating groups. Study evaluation given as percent reached form maximum reachable. Box-and-whisker plots show the median (horizontal line), 25–75th percentiles (box), and the range of data within 1.5 × the IQR (whiskers). Light blue: students of Master course in toxicology (n = 8), salmon-coloured: students of post-graduate course (n = 22), olive-green: experts (n = 6)

The assessment of another high-confidence tier study (AnandaKumar et al. 2025) (Fig. 3 and Table S2) indicate a statistical difference in the study overall assessment between the three groups (p = 0.004).

For this study, the differences in the domains’ assessment are statistically significant (p < 0.05) among the three groups with the exception of “purity and identity” (without Bonferroni correction, Table S2). With Bonferroni correction, the differences in overall assessment and the overall study design remain significant (p < 0.05). Notably, the evaluation of the Master students (median (IQR: 79.1 (75.2–84.4)) closely aligned with those of the experts (median (IQR): 78.8 (72.6–83.3)) whereas the postgraduate group gave lower points compared to the other two groups.

### Exploratory study on the discrimination ability of some AI tools

Results from the exploratory, preliminary assessment (Table 3) demonstrate that the selected AI platforms are capable of completing the questionnaire. Although this initial study suggests that these tools can effectively interpret and respond to the structured requirements of the assessment, the tested tools failed to discriminate between varying levels of internal validity. As seen in Table 3, they struggled to accurately categorize studies across the high, moderate, low, and very low confidence tiers. Generally, the AI models scored studies of low and moderate internal validity higher than the experts. As this was a non-systematic exploratory analysis of available AI models, no statistical comparisons with the experts were performed. Instead, the results are presented to illustrate the responses generated by the AI tools and the corresponding expert assessments.

## Discussion

Systematic review principles have been adopted in chemical risk assessment to analyze animal and human studies. A core component of this process is assessing internal validity, or risk of bias, to detect sources which may bias results of the study. While multiple tools exist for assessing the internal validity of human and animal studies, no specialized framework is currently available to systematically evaluate the internal validity of toxicokinetic studies. Toxicokinetic studies in animals are an integral part of the overall assessment of substances, such as food additives, novel food, chemicals, pesticides, and biocides, for which usually no kinetic information in humans is available. Insofar, a tool for the systematic assessment of a potential risk of bias of the available animal toxicokinetic studies is much-needed.

The group of toxicologists working on the development of the current questionnaire was experienced in assessing toxicological studies in animals and some of them had ample experience in systematically reviewing toxicological animal studies. Special experience in developing and executing analytical methods for substances and also in planning, performing and analyzing kinetic studies was a major asset in constructing the questionnaire.

Generally, in assessing the internal validity of animal toxicity studies, responses are typically integrated via a complex algorithm to categorize studies into three tiers (1 to 3), representing decreasing levels of validity (EFSA FAF Panel 2021; Gundert-Remy et al. 2017; NTP-OHAT 2015, 2019). In this work, we decided to select four tiers in quartiles for the overall assessment and the levels were expressed in levels of confidence: high, moderate, low or very low confidence that the study does not have a high risk of bias. By applying weighting factors to each question, we have accounted for their varying contributions to the study’s overall assessment which increases the transparency of the process. We have also analyzed the points reached in percentages of the maximum attainable number of points (100%) at the domain level to evaluate which domain contributes to which extent to the overall study assessment. This innovative step increases the granularity of the overall assessment outcome. The granular analysis allows identifying the domains with major flaws in the study. Additionally, this approach enables experts to identify high-confidence domains that may still provide valuable toxicokinetic data, even when the study’s overall assessment is rated lower. This ensures that valuable toxicokinetic data can still inform the assessment, provided the associated uncertainty is explicitly documented with a comment on the degree of confidence in the data. In our experience, this is particularly relevant when up-to-date toxicokinetic studies are unavailable and only older studies conducted decades ago are accessible.

The results of the exercise on the discriminatory ability show that the questionnaire is able to separate studies of differing internal validity, leading to a total score between 27% (Howes et al. 1998) and 90% (Kwon et al. 2024). High-confidence studies achieved higher percentage scores across all domains, particularly in Formulation and Sampling and Analysis compared with other studies. This is not astonishing since we noted that the ranking is coincident with the publication year which reflects a chronological improvement in research quality and more stringent analytical and formulation standards (EFSA Scientific Committee 2021; ICH 2022).

The questionnaire was considered understandable by Master students who had limited experience in assessing studies and the same holds true also for the postgraduate course attendees. The wider distribution of scores within the postgraduate group (Figs. 2 and 3) likely reflects their diverse professional backgrounds and varied fields of expertise, whereas the Master students exhibited more consistent results due to the focused orientation of their specialized coursework. Interestingly, experts -having extensive experience- tend to score the tested studies higher which may be attributed to their proficiency in technical terminology and their ability to contextualize reporting deficiencies based on practical experience.

None of the tested Large Language Models were able to discriminate between studies with differing internal validity, at least not with the provided prompt. Since we investigated only a limited number of AI tools, including those which were specifically developed for use in the medical field (e.g. MetaLlama), other AI tools may potentially be better suited for this specific task. However, the selected models represent a strategic spectrum ranging from deep reasoning capabilities (ChatGPT and DeepSeek) to domain-specific expertise (MedGemma and MetaLlama). This may help test the capability of AI to transcend simple text processing to perform the hierarchical judgments required for toxicokinetic validity. In our limited exercise, we could not identify whether AI is currently of support when assessing toxicokinetic studies, especially those of low internal validity.

The primary strengths of our questionnaire lie in its practical application and structured design, which facilitate a highly granular, domain-wise assessment of toxicokinetic data. By presenting results in a tabulated format, the tool ensures ease of interpretation for users regardless of their specific professional background. Beyond its role in systematic reviews, and study screening for risk assessment, the questionnaire supports planning and reporting kinetic studies which may extend also to include animal pharmacokinetic studies. Moreover, it may serve as a standardized benchmark for journal reviewers and authors during manuscript preparation and review.

However, the tool possesses certain logistical limitations, most notably the time required for completion; even experts typically spend at least 30 min per study. To ensure objective results and reach consensus, the current protocol also requires a dual-assessor approach, which may increase the resource burden. Furthermore, the preliminary nature of this validation, specifically with a limited participant pool primarily composed of Master-level students, suggests that further testing with a larger, more diverse cohort of senior experts is required to confirm the tool’s broader reliability. Another limitation of the current study is the fixed nature of the WFs. While WFs were determined based on expert consensus, their fixed nature may introduce potential subjectivity. We did not perform a formal robustness analysis that is common in multi-criterion decision analysis to evaluate how variations in weights might impact the final output. Future research should include sensitivity analyses to ensure the stability of the scoring model across different weighting scenarios.

In conclusion, the newly developed questionnaire for assessing the internal validity of animal toxicokinetic studies has some innovative elements which increase the transparency of the assessment. The procedure is able to discriminate between the internal validity of studies. It offers the possibility to assess a study at the level of overall assessment as well as at the level of the domains performing a granular analysis. The questionnaire can be used with success by experienced toxicologists. However, also toxicologists in training may profit from using the questionnaire.

## Supplementary Information

Below is the link to the electronic supplementary material.

Supplementary Material 1.

Supplementary Material 2.

## Data Availability

The datasets generated during and/or analysed during the current study are available from the corresponding author on reasonable request.
