# Supplementary material for: A tool to assess the internal validity of toxicokinetic studies
Source: Arch Toxicol. 2026 Apr 15;100(7):2955–65. doi: 10.1007/s00204-026-04387-y (PMC13309501; doi:10.1007/s00204-026-04387-y)
Supplement: Supplementary file 2 — Supplementary Material 2. [file 204_2026_4387_MOESM2_ESM.docx]

**Supplementary Information**

**A Tool to Assess the Internal Validity of Toxicokinetic Studies**

Batke M^1#^, Damm G^2^, Foth H^3^, Freyberger A^4^, Hengstler JG^5^, Mangerich A^6^, Mielke H^7#^, Partosch F^8^, Schupp Th^9^, Sonnenburg A^10^, vom Brocke J^11^, Wollin K-M^12^, Algharably EA^13^, Gundert-Remy U^13^

1 Faculty of Chemistry, University of Duisburg-Essen, Essen, Germany

2 Department of Hepatobiliary Surgery and Visceral Transplantation, Leipzig University Medical Center, Leipzig, Germany

3 Institute of Environmental Toxicology, University of Halle, Halle/Saale, Germany

4 Formerly Bayer AG, Wuppertal, Germany

5 Leibniz Research Centre for Working Environment and Human Factors, Dortmund University, Dortmund, Germany

6 University of Potsdam, Nutritional Toxicology Group, Institute of Nutritional Science, Potsdam, Germany

7 Department of Exposure, German Federal Institute for Risk Assessment

8 Ramboll Deutschland GmbH, Essen, Germany

9 University of Applied Sciences Muenster, Steinfurt, Germany

10 Department Pesticides Safety, German Federal Institute for Risk Assessment, Berlin, Germany

11 European Chemicals Agency, Helsinki, Finland

12 Formerly Public Health Agency of Lower Saxony, Hannover, Germany

13 Charité-Universitätsmedizin Berlin, Corporate Member of Freie Universität Berlin and Humboldt Universität zu Berlin, Germany

#These authors have contributed equally to this work

Corresponding author:

Engi Algharably

E-email:

engi.algharably@charite.de

**Table S1 Domain and the overall study assessment for the internal validity of Kwon et al. (2024) using the questionnaire among participating groups**

|  | Master students (n=8) | Postgraduates  (n=22) | Experts  (n=6) | p^a^ |
| --- | --- | --- | --- | --- |
| **Overall assessment** | 72.4 (67.3-78.8) | 68.5 (60.9-73.7) | 86.3 (82.1-93.2) | 0.063 |
| **Overall study design** | 83.3 (77.8-100) | 77.8 (69.4-100) | 100 (83.3-100) | 0.221 |
| **Purity & identity** | 33.3 (33.3-41.7) | 33.3 (16.7-50) | 66.7 (66.7-79.2) | 0.009 |
| **Formulation** | 72.2 (66.7-88.9) | 66.7 (47.2-86.1) | 88.9 (80.6-97.2) | 0.095 |
| **Study conduct** | 84.4 (74.1-92.2) | 71.1 (65-86.1) | 90 (85.6-94.4) | 0.042 |
| **Sampling & analysis^b^** | 68 (59.4-76.8) | 65.2 (61.4-73.7) | 82.8 (75.4-93.1) | 0.171 |

Values are medians (IQR) of percent reached from maximum reachable 100%.

^a^Kruskal-Wallis test.

^b^Combined analysis of domains 5 and 6, i.e. Sample collection and analysis; toxicokinetic analysis and data reporting.

**Table S2 Domain results and the overall assessment obtained by assessing the internal validity of AnandaKumar et al. (2025) using the questionnaire.**

|  | Master students (n=8) | Postgraduates  (n=22) | Experts  (n=6) | p |
| --- | --- | --- | --- | --- |
| **Overall assessment** | 78.8 (72.6-83.3) | 58.8 (49.8-70.5) | 79.1 (75.2-84.4) | 0.004 |
| **Overall study design** | 100 (77.8-100) | 61.1 (44.4-77.8) | 88.9 (77.8-100) | 0.002 |
| **Purity & identity** | 83.3 (62.5-83.3) | 83.3 (54.2-83.3) | 83.3 (58.3-83.3) | 0.830 |
| **Formulation** | 66.7 (63.9-80.6) | 50 (44.4-55.6) | 66.7 (50-66.7) | 0.009 |
| **Study conduct** | 81.1 (70-88.3) | 68.9 (45.2-76.7) | 85.6 (78.3-91.1) | 0.047 |
| **Sampling & analysis** | 81.2 (77.5-85.1) | 52.5 (46.4-78.4) | 81.2 (70.3-90.9) | 0.015 |

Values are medians and 25 to 75 of percent reached from maximum reachable 100%

AnandaKumar SR, Handral M, Seekallu S (2025) Bioavailability study of enantiopure (S)-Equol in CD(SD)IGS rats. Sci Rep 15(1):3141 doi:10.1038/s41598-024-83901-7

Kwon RY, Youn SM, Choi SJ (2024) Oral Excretion Kinetics of Food-Additive Silicon Dioxides and Their Effect on In Vivo Macrophage Activation. Int J Mol Sci 25(3) doi:10.3390/ijms25031614
